# The Aortic Annulus Stabilization Technique Prevents Paravalvular Leaks after Rapid Deployment Aortic Valve Implantation

**DOI:** 10.3390/jcm10245776

**Published:** 2021-12-10

**Authors:** Elena Caporali, Roberto Lorusso, Tiziano Torre, Francesca Toto, Alberto Pozzoli, Giovanni Pedrazzini, Stefanos Demertzis, Enrico Ferrari

**Affiliations:** 1 Department of Cardiology, Cardiocentro Ticino Institute, 8900 Lugano, Switzerland; giovanni.pedrazzini@eoc.ch; 2 Department of Cardiac Surgery, Maastricht University Hospital, 6229 Maastricht, The Netherlands; roberto.lorussobs@gmail.com; 3 Cardiovascular Research Institute Maastricht (CARIM), 6229 Maastricht, The Netherlands; 4 Department of Cardiac Surgery, Cardiocentro Ticino Institute, 8900 Lugano, Switzerland; tiziano.torre@eoc.ch (T.T.); francesca.toto@eoc.ch (F.T.); alberto.pozzoli@eoc.ch (A.P.); stefanos.demertzis@eoc.ch (S.D.); enrico.ferrari@eoc.ch (E.F.); 5 Biomedicine Faculty, Italian Switzerland University (USI), 6900 Lugano, Switzerland

**Keywords:** aortic valve replacement, rapid-deployment aortic valve, annulus stabilization technique, paravalvular leak

## Abstract

Background: Surgical aortic valve replacement with rapid deployment bioprosthesis guarantees good hemodynamic results but carries the risk of paravalvular leaks. To address this issue, an annulus stabilization technique has been recently developed. Methods: Clinical and hemodynamic parameters from patients treated for aortic valve replacement with the rapid deployment bioprosthesis and a concomitant annulus stabilization technique were prospectively collected and retrospectively analyzed. Echocardiographic data at discharge and at 1-year follow-up were collected and analysed. Results: A total of 57 patients (mean age 74.3 ± 6.1 years) with symptomatic aortic valve stenosis underwent aortic valve replacement with the rapid deployment bioprosthesis and concomitant annulus stabilization technique (mean valve size: 23.8 ± 1.9 mm). Combined procedures accounted for 56.1%. Hospital mortality was 1.8% and a new pacemaker for conduction abnormalities was implanted in 10 patients. The pre-discharge echocardiographic control showed absence of paravalvular leaks of any degree in all patients with mean valve gradient of 9.6 ± 4.0 mmHg. The 1-year echocardiographic control confirmed the good valve hemodynamic (mean gradient of 8.0 ± 2.8 mmHg) and absence of leaks. Conclusion: In this preliminary clinical experience, the annulus stabilization technique prevents postoperative paravalvular leaks after rapid deployment aortic valve implantation, up to 1-year postoperatively. Studies on larger series are of paramount importance to confirm the long-term efficacy of this new surgical technique.

## 1. Introduction

Surgical aortic valve replacement (SAVR) with use of biological prosthesis remains the treatment of choice in case of aortic valve disease, reasonable surgical risk, and adequate patient’s age. Recently, the rapid deployment Intuity™ heart valve system (Edwards Lifesciences, Irvine, CA, USA) has been developed in order to improve the surgical implanting time and facilitate the aortic valve replacement in minimally invasive settings [1,2,3]. This bioprosthesis combines the excellent hemodynamic characteristics of the well-known Perimount™ pericardial valve (Edwards Lifesciences, Irvine, CA, USA) with the advantages of an innovative implanting system consisting of a balloon-expanding stent placed below the sewing ring level. The Intuity valve has already shown excellent hemodynamic results in published studies, as well as an extreme versatility, but still carries the risk of postoperative mild to moderate paravalvular leak (PVL), mainly related to the intrinsic characteristics of both the aortic annulus and the fixation system [4,5,6,7]. Therefore, we recently proposed an innovative surgical technique aimed at reducing the risk of PVL following the implantation of the Intuity valve system. This technique consists of a surgical stabilization of the aortic annulus that is performed in concomitance with the aortic valve replacement and allows a better match between the annulus itself and the stent of the Intuity valve [8]. The present study analyses the hemodynamic results and the presence of PVL in a group of patients treated for aortic valve stenosis with the Intuity valve system and concomitant annulus stabilization technique.

## 2. Methods

This is a monocentric non-randomized retrospective study including patients suffering from aortic valve disease (isolated or not) that were operated on for aortic valve replacement with the rapid deployment Intuity valve system and concomitant annulus stabilization technique. Patients signed the informed consent for the surgical procedure and for the treatment of the anonymized clinical data for research purposes. The present investigation abides by the principles outlined in the Declaration of Helsinki (Ethical Principles for Medical Research Involving Human Subjects) adopted by the 18th WMA General Assembly in Helsinki, Finland, June 1964. Ethical review and approval were waived for this study given the retrospective nature of this work.

### 2.1. The Rapid Deployment Intuity Valve System

**Figure 1:**
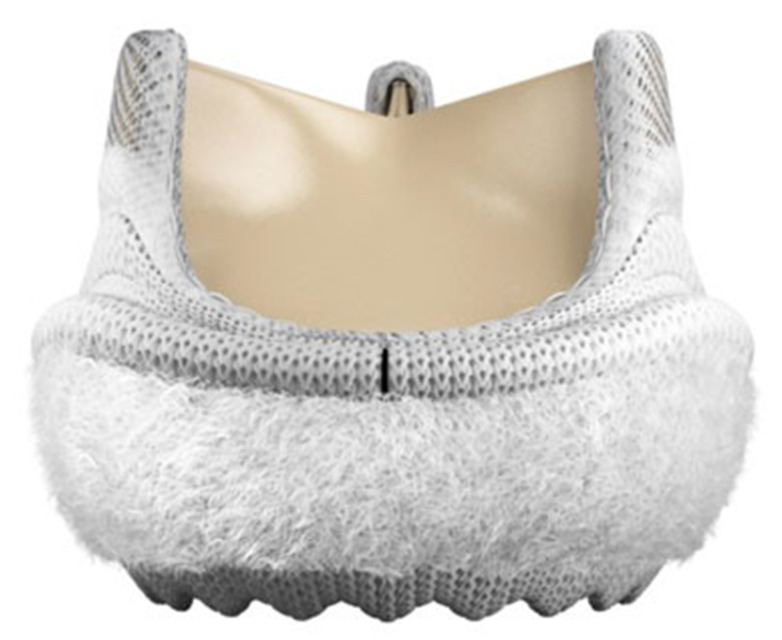
The rapid-deployment Intuity™ aortic valve system (Edwards Lifesciences, Irvine, CA, USA).

The Intuity valve system employed in the present study is the model 8300A from Edwards Lifesciences, available in 5 sizes: 19, 21, 23, 25, and 27 mm. The bioprosthesis is a tri-leaflet stented bovine pericardial valve with a cloth-covered balloon-expanding stent placed below the sewing ring and protruding for 6.6–8.0 mm into the left ventricular outflow tract (Figure 1). The valve requires 3 guiding sutures placed at the nadir of the leaflets’ insertions to the aortic valve annulus, and the nominal balloon inflation pressure required to expand the stent ranges between 4.5 and 5.0 atmosphere according to the manufacturer and depending on the valve size. A layer of low-density polyester cloth envelops the stent to prevent PVL and facilitate the match between the valve and the annulus.

### 2.2. The Annulus Stabilization Technique

**Figure 2:**
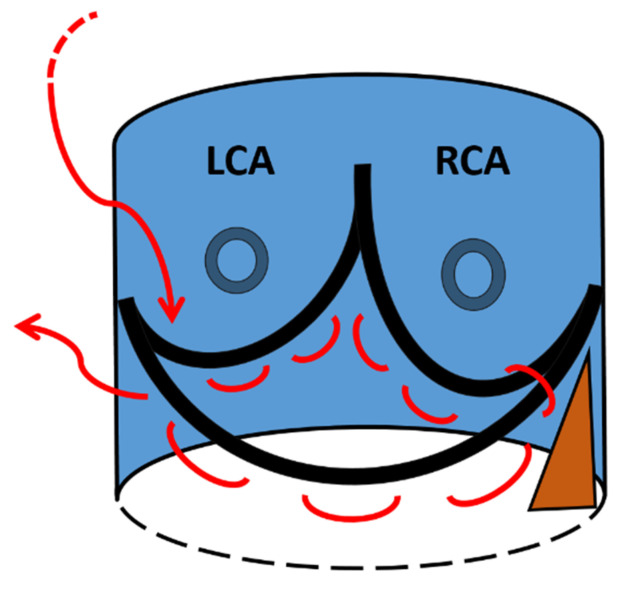
A schematic view of the 3-0 polypropylene purse string suture (red line) seen from the surgeon’s point of view. LCA = left coronary artery; RCA = right coronary artery. The brown triangle identifies the fibrous trigon.

After removing the aortic leaflets and placing the three guiding sutures, a 3-0 polypropylene purse string suture is performed along the bottom edge of the aortic annulus (Figure 2) [8]. The suture starts at left side (surgical view) of the commissure between the non-coronary and the left-coronary cusp and runs, clockwise, 1–2 mm below and parallel to the edge of the aortic annulus. In order to prevent the risk of atrio-ventricular block with subsequent pacemaker implantation, care is taken when the suture approaches the fibrous trigon. In that region, the suture is brought above the annulus and passed behind the commissure. Then, the suture is brought again below the annulus and parallel to the hinge point of the anterior mitral leaflet and finishes at the right side of the commissure between the non-coronary and the left-coronary cusp. At the end of this phase, the purse string suture is not pulled and is left on stand-by. Hence, after inserting the Intuity into the annulus, ballooning the stent, and tightening the 3 guiding sutures, the valve holder is removed and the Intuity is inspected. When the valve is well seated and well-functioning, the purse string suture for the annulus stabilization is pulled and tightened in order to better match the annulus to the stent and prevent paravalvular leaks.

### 2.3. Definitions

Hospital mortality is defined as any death occurring during the hospitalization or within 30 days from the index surgical procedure. Echocardiographic data were collected preoperatively, at discharge, and at one-year follow-up. The degrees of PVL at echocardiographic control were defined as none or trace (not detected or minimal jet (+)), mild (++), moderate (+++), and severe (++++).

### 2.4. Statistical Analysis

Descriptive data were collected, after anonymization, in an Excel spreadsheet (Microsoft Corporation, Cupertino, CA, USA). Continuous variables are described as mean with standard deviation or as median and interquartile range. Dichotomous variables are expressed as absolute number with a percentage of the total.

## 3. Results

From September 2015 to September 2021, 57 consecutive patients with severe symptomatic aortic valve stenosis underwent aortic valve replacement with the Intuity valve system and concomitant annulus stabilization technique. The mean age was 74.3 ± 6.1 years and 35.1% were female patients. Preoperative characteristics are listed in Table 1. The preoperative echocardiogram showed mean left ventricular ejection fraction of 59.7 ± 9.1%, mean aortic orifice area of 0.78 ± 0.2 cm^2^, and mean aortic valve gradient of 42.9 ± 18.4 mmHg.

The surgical and echocardiographic details are listed in Table 2. The procedures were performed either through a full sternotomy (n = 30; 52.6%) or an upper mini-sternotomy in case of isolated SAVR (n = 27; 47.4%). The mean Intuity valve size was 23.8 ± 1.9 mm, the mean aortic cross-clamp time was 71.9 ± 23.4 min, the mean cardiopulmonary bypass time was 95.7 ± 31.4 min, and the mean surgical time was 228.4 ± 63.0 min.

Patients were discharged after a median intensive care unit stay of one day (Interquartile Range 1–3) and a median hospital stay of nine days (Interquartile Range 7–11) (Table 3).

Hospital mortality was 1.8% as one patient died on postoperative day seven for multiple organ failure. In terms of postoperative complications, 10 patients (17.5%) required a permanent pacemaker implantation for intra-cardiac conduction abnormalities and 2 patients (3.5%) experienced neurological disorders (1 TIA and 1 non-disabling stroke completely restored before discharge).

The pre-discharge echocardiographic control confirmed the successful positioning of the rapid deployment Intuity aortic valves, with mean gradient of 9.6 ± 4.0 mmHg and absence of any degree of paravalvular leak in all patients (Table 4). The 1-year echocardiographic follow-up (available for 39 patients) confirmed the good valve hemodynamic with mean gradient of 8.0 ± 2.8 mmHg and absence of any degree of PVL.

## 4. Discussion

This is a preliminary study aiming at analysing the results of a cohort of patients operated for SAVR with the Intuity aortic valve system and concomitant annulus stabilization technique. The major findings are the confirmation of the good Intuity valve hemodynamic at 1-year follow-up and the absence of any degree of paravalvular leak both at postoperative and at 1-year echocardiographic control.

The Intuity valve system has already proven its good hemodynamic characteristics in previously published studies when compared to the well-renowned standard counterpart (the Perimount valve), but there is still a debate when the risk of permanent pacemaker implantation or the presence of paravalvular leaks are concerned [1,2,3,5,6,7,9,10]. In fact, the rapid deployment Intuity valve features a sub-annular balloon-expanding stent that is dilated after the placement of the valve into the annulus. The effectiveness of this anchoring system depends on the intraoperative valve sizing as well as on the characteristics of the aortic annulus when the radial forces of the stent apply against it [7,11,12,13]. In order to reduce the risk of PVL and to anchor the valve, the balloon-expanding stent is intraoperatively ballooned with 4.5 or 5.0 atmospheres, depending on the valve size. However, this step may damage the cardiac conduction system at the level of the fibrous trigon, requiring the implantation of a permanent pacemaker. Moreover, despite the enlargement of the sub-valvular stent, there is still a risk of postoperative PVL, which can lead to an increased mortality rate when moderate or severe, or can cause haemolysis when it is generated by small defects with high-velocity jets [14]. In the TRITON study as well as in other published retrospective studies, the average rate of mild-to-severe postoperative paravalvular leak after Intuity valve implantation still ranges between 1.4% and 13%, and some patients required surgical reoperations in order to address this issue [1,2,3,5,6,7,9,10,11,12,13].

Following these findings, we recently developed a new surgical technique aiming at better matching the aortic annulus to the Intuity valve ring by using a purse string suture running parallel and below the aortic annulus [8]. After a first feasibility study, this is the first clinical report describing a cohort of SAVR patients with the Intuity valve and annulus stabilization technique in which the valve hemodynamic and the echocardiographic results at discharge, and at 1-year follow-up, are recorded and analysed. The results seem to validate the usefulness of the annulus stabilisation technique by showing absence of any degree of PVL in all patients at discharge and after 1-year postoperatively. This is an important achievement suggesting that this technique, easy and reproducible, can be performed in all patients undergoing SAVR with the rapid deployment Intuity valve system.

However, despite the fact that we did not observe complications related to the annulus stabilization technique, we noticed a pacemaker implantation rate of 17.5%, which is slightly higher than the average rate of 5–12.3% reported in previously published papers [2,5,6,7,10,11,15,16,17,18,19]. As already known, the risk of conduction abnormalities is related to the surgical annulus decalcification but also to the stent of the Intuity valve that applies against the conduction system. Therefore, we can imagine that a possible prevention of intra-cardiac conduction abnormalities following the Intuity valve implantation can be a lower inflation pressure for the stent ballooning. This assumption has already been validated in a study from Vogt and co-workers where not the Intuity but a similar valve, the sutureless Perceval valve, was implanted and ballooned at different pressure levels [19,20]. In their study, the balloon post-dilation at a lower pressure, along with the adoption of others technical improvements, was associated with a reduced rate of pacemaker implantation with the Perceval valve. Thus, we can assume that a concomitant annulus stabilization technique can be helpful in preventing PVL with the Intuity valve while the stent is dilated with a lower balloon pressure level, compared to what recommended by the manufacturer. However, in order to confirm this hypothesis, further clinical studies are needed. Another point is that the annulus stabilization technique itself can be a cause of conduction abnormality if the bites of the suture are too deep at septal level. Nevertheless, this is not easy to show, and we suggest the placement of the suture very close to the ventricular rim of the aortic annulus, and not too deep in that region, in order to lower the risk of conduction system damage.

Finally, the annulus stabilization technique can also be useful in case of pure aortic valve insufficiency or aortic valve bicuspidy as these are, so far, contraindications for the use of the Intuity valve system, as higher degrees of PVL are expected. The annulus stabilization technique can also prevent PVL in patients with bicuspidy and pure regurgitation treated with the Intuity valve, and consequently the Intuity can become a bioprosthesis without contraindications for its use; however, this is yet to be demonstrated.

The present study has some limitations as it is a single-centre retrospective study, with a small sample size, no control group, and a short follow-up time. Nevertheless, this is the first report on the clinical use of a new surgical technique aimed at preventing PVL after rapid deployment Intuity valve implantation. In conclusion, further clinical reports with more patients involved are mandatory to demonstrate the effectiveness of this technique, also in bicuspid aortic valves and in pure aortic regurgitations. We also recommend a longer follow-up study to support the long-term effectiveness of this technique in preventing PVL.

## Figures and Tables

**Table 1:** Patients’ characteristics.

Variable	Intuity™ Valve Group (n = 57)
Mean age (years)	74.3 ± 6.1
Female gender	20 (35.1%)
Mean weight (kg)	78.9 ± 16.2
Mean height (cm)	167.0 ± 9.3
Mean BMI	28.1 ± 4.3
Active smokers	13 (22.8%)
COPD	8 (14.0%)
Diabetes Type I	3 (5.3%)
Diabetes Type II	15 (26.3%)
Systemic hypertension	43 (75.4%)
Dyslipidaemia	39 (68.4%)
Chronic renal failure	5 (8.8%)
Coronary disease	27 (47.4%)
Carotid stenosis	3 (5.3%)
Peripheral vascular disease	12 (21.0%)
NYHA Class I	7 (12.3%)
NYHA Class II	26 (45.6%)
NYHA Class III	20 (35.1%)
NYHA Class IV	4 (7.0%)
Euroscore II	3.48 ± 4.3
Mean LVEF (%)	59.7 ± 9.1
Mean aortic valve area (mm^2^)	0.78 ± 0.2
Aortic peak gradient (mmHg)	68.8 ± 27.7
Aortic mean gradient (mmHg)	42.9 ± 18.4

Data are presented as mean ± SD or N (%). BMI, body mass index; COPD, chronic obstructive pulmonary disease; NYHA, New York Heart Association; LVEF, left ventricular ejection fraction.

**Table 2:** Intraoperative data.

Variable	Intuity™ Valve Group (n = 57)
Full sternotomy	30 (52.6%)
Upper mini-sternotomy	27 (47.4%)
Mean valve size (mm)	23.8 ± 1.9
21 mm	11 (19.3%)
23 mm	20 (35.1%)
25 mm	19 (33.3%)
27 mm	7 (12.3%)
Isolated AVR	27 (47.4%)
Concomitant ascending aorta replacement	2 (3.5)
Concomitant coronary surgery	24 (42.1%)
Concomitant mitral valve surgery	4 (7.0%)
Cardiopulmonary bypass time (min)	95.7 ± 31.4
Isolated AVR	73.1 ± 16.4
Combined AVR	113.3 ± 29.0
Aortic cross clamp time (min)	71.9 ± 23.4
Isolated AVR	55.3 ± 11.4
Combined AVR	84.9 ± 22.2
Operating time (min)	228.4 ± 63.0
Isolated AVR	187.8 ± 50.0
Combined AVR	260.2 ± 53.6

Data are presented as mean ± standard deviation or N (%). AVR, aortic valve replacement.

**Table 3:** Outcome.

Variable	Intuity™ Aortic Group (n = 57)
Acute kidney failure	7 (12.3%)
Myocardial infarction	0 (0%)
Non-disabling stroke	1 (1.8%)
TIA	1 (1.8%)
New onset of atrial fibrillation	18 (31.6%)
New pacemaker implantation	10 (17.5%)
Bradycardia (junctional rhythm)	3 (5.3%)
Bradycardia with atrial fibrillation	2 (3.5%)
Bradycardia with trifascicular block	1 (1.7%)
Third-degree atrioventricular block	4 (7.0%)
Wound infections	0 (0%)
Hospital mortality	1 (1.8%)
Cause of death	Multiple organ failure
ICU stay, days (median, IQR)	1 (1–3)
Hospital stay, days (median, IQR)	9 (7–11)

Data are presented as N (%) or median with interquartile range (IQR). TIA, transient ischemic attack; ICU, intensive care unit.

**Table 4:** Echocardiographic data.

Variable	Preoperative (n = 57)	Discharge (n = 57)	1-Year Follow-Up (n = 39)
Peak gradient (mmHg)	68.8 ± 27.7	17.6 ± 7.5	14.5 ± 5.1
Mean gradient (mmHg)	42.9 ± 18.4	9.6 ± 4.0	8.0 ± 2.8
LVEF (%)	59.7 ± 9.1	57.8 ± 8.0	58.2 ± 6.7
Paravalvular leak			
0 or Grade I (trivial)	-	57	39
Grade II (mild)	-	0	0
Grade III (moderate)	-	0	0
Grade IV (severe)	-	0	0

Data are presented as mean ± standard deviation or N (%). LVEF, left ventricular ejection fraction.

## Data Availability

All data are available at Cardiocentro Ticino Institute.
